# Physicochemical and microbiological evaluation of antioxidant-rich traditional black carrot beverage: Kanji

**DOI:** 10.1186/s42269-021-00594-y

**Published:** 2021-08-10

**Authors:** Chetna Sharma, Param Pal Sahota, Sarabjit Kaur

**Affiliations:** Department of Microbiology, Punjab Agricultural University, Ludhiana, Punjab 141004 India

**Keywords:** Black carrots, Fermentation, Functional foods, Lactic acid bacteria, Nutraceuticals, *Pediococcus*

## Abstract

**Background:**

*Daucus carota* subsp. *sativus* (Black carrots) are underutilized in context to their nutritional properties. In this study, Kanji, a naturally fermented probiotic beverage, was prepared using *Daucus carota* subsp. *sativus* (var. *Punjab Black Beauty*). Analysis of the physicochemical and microbiological profile of the naturally fermented Kanji was investigated to boost its utilization for commercial purpose.

**Results:**

The physicochemical parameters observed in the fermented Kanji beverage were pH 3.47, total soluble solids 3°B, lactic acid 0.99%, total sugars 36.32 mg/mL, total reducing sugars 27.16 mg/mL, flavonoids 38.14 mg/mL, phenols 40.8 mg/mL, antioxidant activity 79.96% and ascorbic acid 110 mg/100 mL. The microbiological analysis revealed an exponential increase in lactic acid bacterial count from 3.96 to 8.33 log CFU/mL. Out of 11 bacterial strains isolated from Kanji, the bacterial strain with high growth potential was genotypically characterized as *Pediococcus acidilactici* with accession number MK028218.

**Conclusion:**

The lactic acid bacterial Kanji beverage was characterized as a potential plant-based probiotic with high antioxidant activity. This functional autochthonous starter from the Kanji can be used for selective fermentation of black carrots for Kanji ascertaining its microbiological safety, unique flavor and aroma, and consumption as a commercial non-dairy-based probiotic beverage.

## Background

Fermentation, from the ages, serves as a food processing method harnessing microorganisms, primarily lactic acid bacteria (LAB), and yeast. In contrast with their unfermented counterparts, consumers prefer fermented products because of their characteristic aroma, texture, taste, and color (Rice-Evans et al. 1997). Probiotic food products are in concatenation with such foods and are viable selective microbial dietary supplements provided in an appropriate amount to bestow health benefits apart from the general nutrition. The impact on human health by probiotic lactic acid fermented foods has been promptly suggested by health and medical professionals (Perricone et al. 2010). Probiotics, by definition, are “viable single or mixed bacterial preparation which, when provided to the host, benefits them by enhancing the properties of the gut flora” (In't Veld et al. 1994). Probiotics are required to be taken regularly in adequate doses (≥ 10^6^ CFU/mL daily) to avoid washout and ensuring the benefits are acquired in a sustained manner.

Most probiotic foods were constricted to dairy products but nowadays, plant-based fermented products are being increasingly considered as vectors for probiotic delivery (Soccol et al. 2010). Significant number of lactose intolerant people, higher cholesterol levels of dairy foods, and pronounced economic reasons in various developing countries necessitate the seek out for the alternatives with higher nutritive value along with health-promoting factors, e.g., vegetables, fruits, cereals, and legumes, etc. (Perricone et al. 2010) and from products which have lower cholesterol content nevertheless rich in protein, starch, minerals, fibres, vitamins, and antioxidant contents. Besides this, with the popularism of vegetarian diets in both developed and developing countries, the demand for plant-based probiotics has elevated significantly. A large number of non-dairy-based traditionally fermented products are consumed all over the globe. However, these products have not been exploited commercially as probiotic beverages due to a lack of required scientific research. Plant-based fermented products are preferred on account of their bioactive properties along with their wholesome nutrient profile and unique taste and flavor.

Anthocyanin-rich black carrots (*Daucus carota* subsp. *sativus*) cultivated mainly in Northern India are utilized in the preparation of the lactic acid fermented probiotic beverage ‘Kanji’ by spontaneous fermentation of uniformly grated black carrots with requisite condiments concentration. Black carrots are in the focus due to their high anthocyanin content and extraordinary quality parameters (Kirca et al. 2006). Kanji beverage exhibits diuretic, digestive tract soothing, hepatoprotective, and uterine stimulating potential. The major anthocyanins in these carrots are derived from the acylation of cyanidins (Rice-Evans et al. 1997) and have greater color stability at food pH in contrast to the other anthocyanin sources of plant origin. Studies also suggest that LAB strains from Kanji showed their potential as probiotics and showed acid-bile salt tolerance, cholesterol assimilation, and antimicrobial activity against food-borne pathogens (Lamba et al. 2019). The identified strains were K1a: *Lactobacillus curvatus, Lactobacillus delbrueckii*, and K23c: *Lactobacillus coryniformis* (Reddy et al. 2007). Furthermore, *Lactobacillus plantarum* isolated from Kanjika was evaluated as a potential source of Vitamin B12 (Madhu et al. 2010).

The autochthonous LAB varies as a function of the quality of the raw material, harvesting conditions, and temperature. It was shown that the use of a starter culture helps to standardize the fermentation by controlling the microbial flora (Font de Valdez et al. 1990). This study was undertaken to study the nutritional profile of Kanji beverage and the isolation and characterization of the lactic acid cultures (LAB) present during fermentation from their natural environment. This study can be the basis for the development of potential starter cultures with predictable characteristics which can be used for commercial production of Kanji with consistent quality and good nutritive profile.

## Methods

### Fermentation process

Black carrots (*Daucus carota* subsp. *sativus*) of variety *Punjab Black Beauty* were procured from the Department of Vegetable Sciences, PAU, Ludhiana, Punjab, India. Common salt and rye as adjuvants were purchased from the local market, Ludhiana, Punjab. Fresh black carrots (3 kg) were washed with lukewarm water containing 0.01% potassium metabisulfite (KMS) solution for surface sterilization. The calyx was removed and carrots were aseptically lightly peeled. 3 L of boiled and then cooled bacteriologically safe water was added to 1 L of black carrot juice. Condiments salt and rye (60 g each) were added to the fermenter after roasting them in the microwave oven at 800 watts for 1 min. No starter culture was added as natural autochthonous flora was allowed for the spontaneous fermentation of the Kanji beverage. Fermentation of black carrot beverage was allowed at room temperature (RT) for five days which ensures the establishment of lactic acid bacteria in the beverage and reduces the risk of contamination. The naturally fermented Kanji beverage was stored at RT for six months in sterilized glass bottles. The bottles were sterilized by autoclaving. Microbiological and physicochemical analyses were performed at an interval of 20 days.

### Microbiological analysis of naturally fermented Kanji

The traditionally fermented Kanji samples were diluted serially in 0.9% autoclaved saline and inoculated onto de Man Rogosa and Sharpe (MRS) agar plates and were incubated at 37 °C for 24–48 h for total lactic acid bacterial counts. For the count of total mesophilic aerobic bacteria (TMAB), plate count agar was used with incubation at 37 °C for 24–48 h. Enumeration of total yeast count was performed using potato dextrose agar and plates were incubated at 28 °C for three days. The total coliform count was performed by sample enumeration on crystal violet neutral red bile lactose agar (VRBL agar) at 37 °C for 24–48 h.

### Biochemical and genotypic characterization of bacterial strains

The biochemical and phenotypic characteristics of the lactic acid strains were compared with the previously published data for the identification of the cultures. Bacterial isolate with high growth potential was further analyzed by molecular characterization. After isolating Genomic DNA using the Schubert method (Schubert et al. 2008), the forward and reverse primers 27F (5′AGAGTTTGATCMTGGCTCAG3′) and 1492R (5′TACGGYTACCTTGTTACGACTT3′) were used in the amplification of the 16S gene. The PCR reaction was carried out in 100 μL volume (10 μL 10 × PCR buffer, 2.5 μL dNTPs mix, 2.5 μL each primer, 1 μL Taq DNA polymerase) with 3 μL DNA template. The PCR cycle (Peltier Thermal Cycler, BIORAD) was conducted with the following program: 95 °C for 5 min, 30 cycles of 1 min at 95 °C, 1 min at 55 °C, 1 min at 72° and 5 min at 72 °C. The PCR products were resolved by gel electrophoresis using 1.5% agarose gel and visualized by ethidium bromide staining. These PCR products were sent to a sequencing company for identification (Eurofins Genomics India Pvt Ltd, Karnataka, India) and the 16S rDNA sequences were compared with known sequences in Genbank using BLAST (http://www.ncbi.nlm.nih.gov/blast). The 16S rRNA gene sequences have been submitted to NCBI GenBank Database.

### Physicochemical analysis of naturally fermented Kanji

#### pH, titrable acidity (TA), total soluble solids (TSS), and sugar content

pH and TA were analyzed according to standard protocols (AOAC 1984). TSS was measured by Erma hand Refractometer of 0–32°Brix (UNICO). Total sugars and reducing sugars were measured using Dubois et al. (1956) and Miller (Lamba et al. 2019) method, respectively.

#### Flavonoids and phenols content

Flavonoids (mg Quercetin equivalents) were evaluated by the method given by Chang et al. (2002) with small modifications. An aliquot of 200 µL of each sample was taken to make up the final 1 mL volume using methanol. 100 µL 10% aluminium nitrate solution was then added to each test tube. Then, 100 µL of 1 M potassium acetate was added followed by the addition of 4.6 mL distilled water. All samples were vortexed thoroughly and kept at room temperature for 45 min. Finally, the absorbance (OD_415_) was measured against a reference blank prepared in methanol.

Phenol content (mg Gallic acid equivalents) was investigated by the method of Singleton and Slinkard (1977) with slight modifications. 200 µL of each sample/ standard was taken and 1 mL volume was made using methanol. Then, 2 mL of diluted Folin-ciocalteu reagent (1:9) was added and the contents were mixed thoroughly. 2 mL of 15% Na_2_CO_3_ solution was added after 4 min and was kept at RT for 2 h. In the end, the absorbance (OD_760_) of the samples was measured.

#### Antioxidant activity (%) by DPPH assay

% Antioxidant activity was measured by DPPH method (Brand-Williams et al. 1995). For estimation, a 100 µL sample was taken and 3.9 mL DPPH solution (1 μM DPPH) was added afterward. Samples were placed in the dark for 45 min. Discoloration in the solution was measured at wavelength 515 nm using Bausch & Laumb Spectronic-20. Ascorbic acid was used as the standard for the calculations. % Antioxidant activity was calculated by the formula given below:

$$\%{\text{ Antioxidant Activity}} = \left[ {1 - \frac{{\text{Absorbance of sample}}}{{\text{Absorbance of standard}}}} \right] \times 100$$

#### Ascorbic acid content

2, 6-Dichlorophenol indophenol dye titration method was used in the estimation of ascorbic acid (AOVC 1996). Ascorbic acid content was calculated by the given formula:

$${\text{mg of Ascorbic acid/}}100\;{\text{ml}} = \frac{{{\text{Titre value}} \times {\text{Dye factor}} \times {\text{Volume made}}}}{{{\text{Aliquot taken}} \times {\text{Weight of sample}}}} \times 100$$

### Statistical analysis

The experiment was performed in triplicates. The data shown are means (± SEM) of triplicates for each observation. The data were analyzed by one-way ANOVA in completely randomized design (CRD) using SAS software, version 9.4. Tukey’s multiple comparison test (*α* = 0.05) was used for examining significant differences between various observations.

## Results

### Enumeration of microorganisms in fermented Kanji

**Fig. 1:**
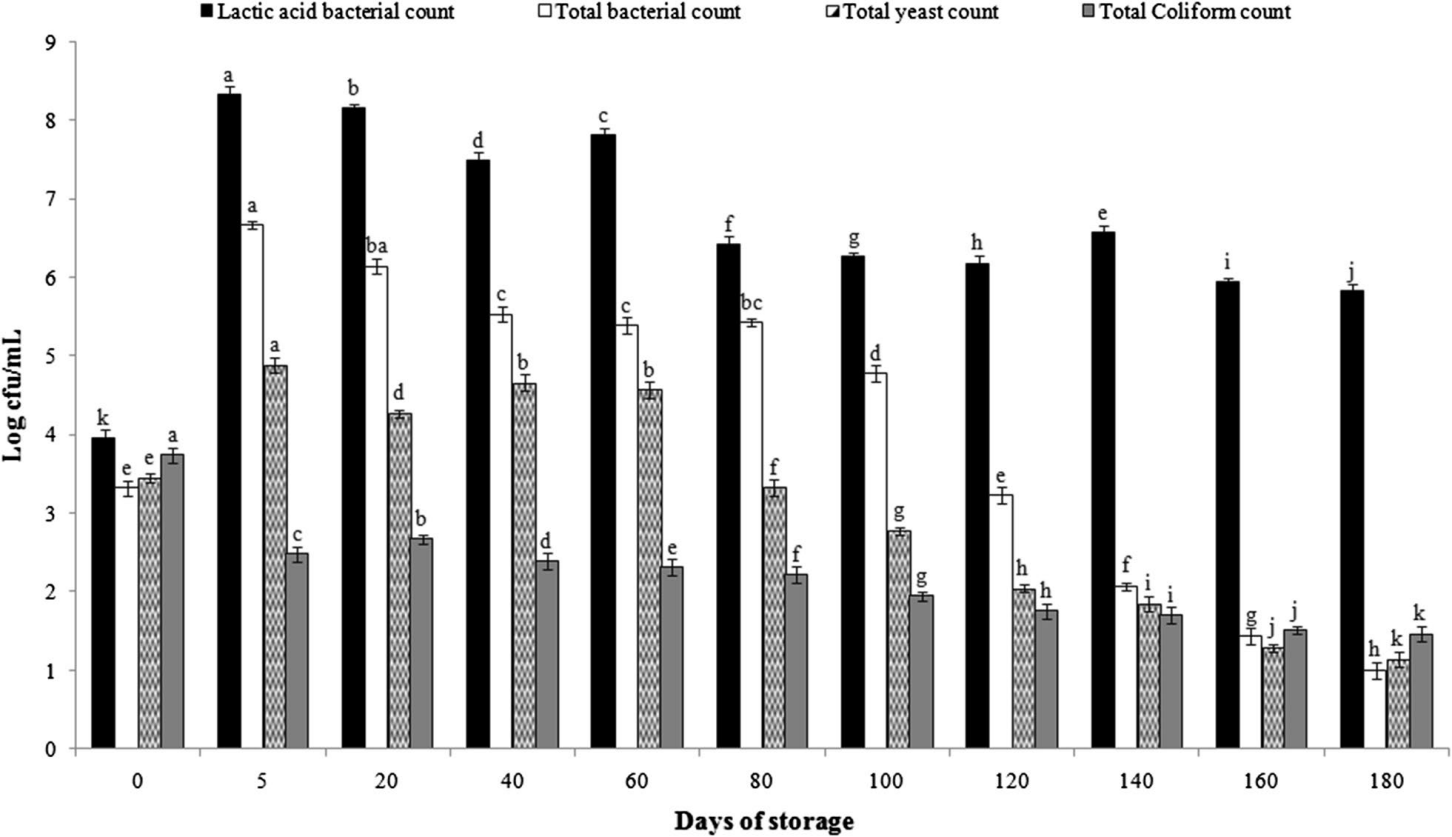
Changes in lactic acid bacterial and total bacterial count during 6 months in Kanji beverage. *All observations are average of triplicates. **Means with the same letter are not significantly different

Viable microbial counts analyzed in the Kanji beverage during six months are shown in Fig. 1. Total viable bacterial and LAB count was 3.32 log CFU/mL and total lactic acid bacterial count 3.96log CFU/mL at the beginning of Kanji fermentation. The LAB viable count reached a maximum (8.33 log CFU/mL), thereafter slightly declined to 8.15 log CFU/mL after 20 days. These high LAB counts in the Kanji could be due to nutrient-rich Black carrots. Similarly, high levels of LAB counts between 7.1 and 8.90 log CFU g^−1^ were reported (Tanguler and Erten 2012). Instead of the classical stationary phase after maximum growth, LAB decreased during storage. The lactic acid bacterial count was recorded 5.82 log CFU/mL at the end of six months which was a little lesser than the minimum value recommended of 10^6^ cfu/mL of viable cells for probiotic products. The cell viability of lactic acid bacteria is decisive for the stability and quality of the fermented product.

### Biochemical and genotypic characterization of bacterial isolates

Initially, 11 bacterial strains were selected from the Kanji beverage based on morphological and biochemical characterization and were preserved at 4 °C. These inherent cultures are autochthonous in the Kanji beverage and survive without the need for any supplement or nutrient. Out of these 11, seven isolates were labeled as PAU1, PAU3, PAU4, PA5, PAU7, PAU9, and PAU11 and were morphologically and biochemically characterized as shown in Table 1. Bacterial isolate PAU11 was genotypically identified as *Pediococcus acidilactici* and was submitted to the Genbank, NCBI database with accession number MK028218. Isolation of *Pediococcus* sp. in the black carrot beverage Shalgam was reported (Tangüler and Erten 2013) but no published data is found on the isolation of *Pediococcus acidilactici* from black carrot beverage.

**Table 1:** Morphological and biochemical characterization of bacterial isolates

	PAU1	PAU3	PAU4	PAU5	PAU7	PAU9	PAU11
Gram reaction	+ Long rods	+ Rods	+ Cocci in pairs	− Short rods	− Short rods	− Cocci	+ Cocci
Colony characteristic	Large, wrinkled, creamish, oval	Creamish, irregular and slimy	Small, colorless and chalk	Yellow pigmented, round	Raised, white, mucoid	Small, colorless and pinpointed	Small, white and round
Biochemical characteristics							
Sugar fermentation							
Maltose	+	+	+	+	+	+	+
Lactose	+	+	+	+	−	+	+
Galactose	+	+	+	+	+	+	+
Raffinose	+	+	+	+	+	+	+
Cellobiose	+	+	+	+	+	+	+
Mannose	+	+	+	+	+	+	+
Trehalose	+	+	+	+	+	+	+
Citrate utilization	+	+	+	+	−	+	+
Triple Sugar Iron (TSI) agar	+A −G	+A −G	+A −G	+A −G	+A −G	+A −G	+A −G
Nitrate reduction	−	−	−	−	−	−	−
Esculin hydrolysis	+	+	+	+	+	+	+
Urease	−	−	−	−	−	−	−
ONPG	−	+	−	−	+	−	−

PAU (in isolate name) used as a label and signifies Punjab Agricultural University

### Physicochemical analysis of fermented Kanji

#### Analysis of pH, TA, TSS, and sugar content

pH, TA, TSS, and sugar content of the Kanji beverage are shown in Table 2. There was a significant reduction in the pH and an increase in TA. The initial pH 6 and TA 0.21% were recorded 3.47 and 0.99% were recorded after fermentation. Reduced pH and increased TA are of immense importance to the quality of the beverage (Viander et al. 2003). pH and TA range was recorded 3.47–3.78 and 0.98–1.08% during the storage period of 180 days. These results were in good agreement with the findings of the traditionally fermented Shalgam beverage (Tanguler and Erten 2012). Reduced pH and high TA recorded during the storage could be contributing factors for the shelf stability of the Kanji beverage.

**Table 2:** Effect of storage on the physicochemical parameters of the naturally fermented beverage

Days of storage	pH	TSS (°Brix)	% TA	Total sugars (mg/mL)	Reducing sugars (mg/mL)	%Antioxidant capacity	Flavonoids (mg/mL)	Phenols (mg/mL)	Ascorbic acid (mg/100 mL)
0	6 ± .0.02^a^	6.5 ± 0.02^a^	0.21 ± 0.05^b^	78.21 ± 0.2^a^	39.79 ± 0.02^a^	70.75 ± 0.03^a^	33.2 ± 0.01^a^	33.5 ± 0.01^f^	94 ± 0.02^ba^
5	3.47 ± 0.01^h^	3 ± 0.05^c^	0.99 ± 0.02^a^	36.32 ± 0.1^e^	27.16 ± 0.01^cb^	79.96 ± 0.02^a^	38.14 ± 0.02^a^	40.8 ± 0.02^a^	110 ± 0.03^a^
20	3.49 ± 0.01^hg^	2.8 ± 0.02^d^	0.98 ± 0.01^a^	36.11 ± 0.2^e^	26.21 ± 0.02^cd^	82.46 ± 0.01^a^	40.02 ± 0.01^a^	39.8 ± 0.02^ba^	110 ± 0.02^a^
40	3.51 ± 0.02^g^	3 ± 0.02^c^	1.04 ± 0.02^a^	36.16 ± 0.05^e^	26.95 ± 0.02^cb^	80.79 ± 0.01^a^	36.87 ± 0.02^a^	39.7 ± 0.02^bac^	108 ± 0.01^a^
60	3.62 ± 0.01^f^	2.8 ± 0.03^d^	1.05 ± 0.02^a^	36.37 ± 0.1^e^	27.47 ± 0.02^cb^	79.98 ± 0.005^a^	36.72 ± 0.02^a^	38.8 ± 0.02^bdc^	102 ± 0.02^ba^
80	3.71 ± 0.01^e^	3 ± 0.02^c^	1.06 ± 0.02^a^	37.89 ± 0.2^d^	27.89 ± 0.02^b^	77.56 ± 0.02^a^	36.23 ± 0.02^a^	38.2 ± 0.01^edc^	97 ± 0.01^ba^
100	3.75 ± 0.01^d^	2.8 ± 0.02^d^	1.04 ± 0.02^a^	38.05 ± 0.02^cd^	26.95 ± 0.01^cb^	76.15 ± 0.02^a^	35.04 ± 0.02^a^	38.4 ± 0.02^bedc^	88 ± 0.005^bac^
120	3.76 ± 0.01^cd^	3 ± 0.02^c^	1.05 ± 0.01^a^	38.53 ± 0.2^cb^	26.11 ± 0.02^d^	74.31 ± 0.02^a^	35.78 ± 0.02^a^	38.7 ± 0.03^ed^	85 ± 0.03^c^
140	3.79 ± 0.01^b^	2.8 ± 0.02^d^	1.07 ± 0.02^a^	38.66 ± 0.1^b^	26.63 ± 0.01^cbd^	73.81 ± 0.02^a^	35.87 ± 0.02^a^	37.9 ± 0.02^ed^	83 ± 0.05^bc^
160	3.78 ± 0.02^cb^	3.2 ± 0.02^b^	1.08 ± 0.01^a^	38.84 ± 0.1^b^	26.49 ± 0.02^cbd^	73.24 ± 0.02^a^	34.07 ± 0.01^a^	37.7 ± 0.005^ed^	80 ± 0.01^bc^
180	3.78 ± 0.01^cb^	3.2 ± 0.01^b^	1.06 ± 0.03^a^	38.84 ± 0.2^b^	26 ± 0.2^cd^	72.78 ± 0.01^a^	34.04 ± 0.01^a^	37.07 ± 0.02^e^	80 ± 0.02^bc^

*TA* titrable acidity, *TSS* total soluble solids

All observations are average of triplicates

Means with the same letter are not significantly different

Initial TSS 6.5 reduced to 3 after 5 days of natural fermentation. Throughout the storage, it remained in the range 2.8–3.2. Total sugars and reducing sugars were recorded 78.21 and 39.79 mg/mL initially and reduced significantly to 36.32 and 27.16 mg/mL after natural fermentation. However, these residual sugars were present in the Kanji during storage. Total sugars and reducing sugars were recorded 38.84 and 26 mg/mL after six months of storage.

#### Analysis of flavonoid and phenol content

Phenolics and flavonoids, the secondary metabolites, occur throughout the plant kingdom. Phenols have an antioxidative effect by interacting with phenol ring and its resonance stabilization effect (Panghal et al. 2017) and flavonoids are available mostly in complexed forms such as o-glycosidic with several sugars such as glucose, galactose, arabinose, rhamnose, rutinose, and xylose. The levels of phenolics and flavonoids in the Kanji tested were significantly higher 40.8 mg/mL and 38.14 mg/mL in contrast to 33.5 mg/mL and 33.2 mg/mL of unfermented preparation (Table 2). This increase could be due to the enzymatic action and acid production of the strains facilitating the release of flavonoids and phenols from their complex form into more soluble free form Katina et al. (2007). At 3-month interval, phenolics and flavonoids were recorded 38.8 and 36.72 mg/mL and were 37.07 and 34.04 mg/mL after 6 months of storage. The phenolic and flavonoid content was higher than the unfermented preparation throughout the storage period.

#### % Antioxidant capacity

1, 1-Diphenyl-2-picryl hydrazyl (DPPH) antioxidant activity was measured in terms of percentage scavenging of a pre-formed free-radical by antioxidants in the samples. The antioxidant capacity of kanji increased after fermentation and was found 79.96% in Kanji (Table 2). This can be associated with the presence of phytochemicals like flavonoids and phenolics in Kanji which are an excellent source of antioxidants (Kikuzaki et al. 2002). The highest 82.46% antioxidant capacity was recorded at 20 days of storage. In a previous study, Haria fermented with *L. fermentum* KKL1 also showed a strong 82.54% antioxidant capacity against DPPH (Ghosh et al. 2015).

#### Ascorbic acid content

The ascorbic acid content of traditionally fermented Kanji is shown in Table 2. The ascorbic acid content of the unfermented beverage recorded was 90 mg/100 mL. A significant increase to 100 mg/100 mL was recorded after fermentation which was stable up to the 40th day of storage, thereafter, gradually reduced. At the end of 6 months, ascorbic acid was recorded 80 mg/100 mL in the fermented Kanji. Reduction in ascorbic acid content during storage could be due to the oxidation activity of enzyme ascorbic acid oxidase by the collective effect of light and oxygen (Bhardwaj and Mukherjee 2011). Daily intake of ascorbic acid or vitamin C is, however, very popular nowadays. Appropriate doses of ascorbic acid (Vitamin-C) can be a preventive approach for the novel coronavirus, COVID-19 (Wang et al. 2020). Studies suggest that ascorbic acid may prevent the receptiveness of lower respiratory tract infection (Hemila 1997), while COVID-19 may cause lower respiratory tract infection.

## Discussion

Based on the nutritive analysis, Kanji holds a promising alternative for dairy-based probiotics and act as a plant-based probiotic delivery vehicle. Carrot juice serves as a good dairy alternative growth medium for probiotic growth. Carrot juice constitutes of 2% (w/v) sucrose, 1% (w/v) glucose, and 0.8% (w/v) fructose (Kun et al. 2008). Glucose and sucrose are the major sources of energy and carbon for the growth of lactic acid bacteria in carrot juice. Both *L. rhamnosus* and *L. bulgaricus* exhibited significant growth up to about 10^9^ cfu/mL at the end of fermentation in carrot juice (Nazzaro et al. 2008). However, there is no report of the previous occurrence of *Pediococcus acidilactici* in the Kanji fermentation. For probiotic preparations, there are yet not any fixed standards, nonetheless, the US FDA recommendation of the minimum probiotic count level is at least 10^6^ cfu/mL in a probiotic food. Taking into account the ingested amount and effect of various storage conditions on probiotic viability, 10^8^–10^9^ probiotic microbial daily intake is crucial to achieve probiotic action in human (Knorr 1998). It has also been suggested that probiotics should be consumed on regular basis (approximate consumption: 100 g) to be effective in delivering about 10 g viable cells into the instestine (Miller 1959). During the storage period of 60 days*,* the count reached approximately 10^8^ CFU/mL level which suggests exploring the probiotic potential of the strain. Yeast and Coliform count decreased in the Kanji beverage after fermentation. Low pH and the addition of condiments may result in the decrease of coliform and yeast count due to their antimicrobial properties (Madhu et al. 2010). Salt added in the juice does not exhibit antimicrobial activity, but it reduces the water activity in foods which results in the slowing down or interruption in the vital microbial processes and hence increases the shelf stability of a product. Salt serves as a preservative during the fermentation of fruits and vegetables. The Kanji beverage prepared was safe for consumption according to food standards (Food Safety and Standards Authority of India 2018).

The high viable cell counts suggest that carrot supplied sufficient nutrients for the multiplication for all the bacterial strains. Besides that the presence of phenolic compounds and antioxidants could have contributed for a higher survival through the creation of anaerobic conditions in ideal proportions that favored the multiplication of probiotic microflora (Lima et al. 2012). The high levels of the residual sugars in Kanji suggest the role of pH to stop the fermentation process contrary to the lack of carbohydrate substrate required and results were in agreement with study of Gardner et al. (2001) which reported dual utilization of glucose and fructose and high quantity of residual sugars as feature of LAB metabolism. Furthermore, the high antioxidant activity in the beverage resulted due to the increase in the phenols and flavonoids. The increase in flavonoids content may have resulted due to the increase in acidic metabolites during fermentation, which involves unchaining bound flavonoids components and converting them into available form Ademiluyi and Oboh (2011). In context to this, phenolic compounds in natural medium are associated with sugar, which decreases their availability. During fermentation process, proteases hydrolyze complexes of phenolics into soluble-free phenols and other simpler and biologically more active that can be readily absorbed (Ademiluyi and Oboh 2011). The evaluation of antioxidant activity in food products has an important role in the nutritional research as it provides constructive information regarding the functional quality of food material without the analysis of each antioxidant compound (Scalfi et al. 2000).

## Conclusion

Consumer demand for functional non-dairy-based products has been increased and probiotics are being incorporated to produce ready-to-serve drinks with plant-based origin. Despite their high nutritional content, black carrots (*var. Punjab Black Beauty*) are under-utilized due to their short shelf life and flavor preference. Kanji serves as an alternative to exploit highly nutritive minimally processed black carrots providing health benefits to the consumer. The isolated lactic acid bacterial strain *Pediococcus acidilactici* possesses desirable probiotic properties and can serve as novel autochthonous starter cultures for fermentation studies to assess their technological characteristics. *Pediococcus sp*. has the functional property of bacteriocin production which can enhance the safety of the beverage prepared. The results obtained underlined that the presence of the tested probiotic strain in the fermented black carrot beverage was well supported. However, the probiotic delivery using food substrate often needs special technologies. Based on the present results, the development of bioprocess for microbiologically safe and controlled fermentation method with selective autochthonous functional starter cultures for production of a shelf-stable highly nutritive Kanji beverage is required. Moreover, the development of probiotic black carrot product helps to sustain the rural economy by the development of small-scale industries of agriculturists with minimal processing cost.

## Data Availability

Not applicable.

## Abbreviations

FSSAI: Food Safety and Standards Authority of India
FDA: Food and Drug Administration
LAB: Lactic acid bacteria
NCBI: National Centre for Biotechnology Information
TA: Titrable acidity
TSS: Total soluble solids
PAU: Punjab Agricultural University
